# Analysis of the Differentially Expressed Proteins in Donkey Milk in Different Lactation Stages

**DOI:** 10.3390/foods12244466

**Published:** 2023-12-13

**Authors:** Miaomiao Zhou, Fei Huang, Xinyi Du, Guiqin Liu, Changfa Wang

**Affiliations:** School of Agricultural Science and Engineering, Liaocheng Research Institute of Donkey High-Efficiency Breeding and Ecological Feeding, Liaocheng University, Liaocheng 252000, Chinawangchangfa@lcu.edu.cn (C.W.)

**Keywords:** donkey milk, protein, biological activity, lactation stage

## Abstract

Proteins in donkey milk (DM) have special biological activities. However, the bioactive proteins and their expression regulation in donkey milk are still unclear. Thus, the differentially expressed proteins (DEPs) in DM in different lactation stages were first investigated by data-independent acquisition (DIA) proteomics. A total of 805 proteins were characterized in DM. The composition and content of milk proteins varied with the lactation stage. A total of 445 candidate DEPs related to biological processes and molecular functions were identified between mature milk and colostrum. The 219 down-regulated DEPs were mainly related to complement and coagulation cascades, staphylococcus aureus infection, systemic lupus erythematosus, prion diseases, AGE-RAGE signaling pathways in diabetic complications, and pertussis. The 226 up-regulated DEPs were mainly involved in metabolic pathways related to nutrient (fat, carbohydrate, nucleic acid, and vitamin) metabolism. Some other DEPs in milk from the lactation period of 30 to 180 days also had activities such as promoting cell proliferation, promoting antioxidant, immunoregulation, anti-inflammatory, and antibacterial effects, and enhancing skin moisture. DM can be used as a nutritional substitute for infants, as well as for cosmetic and medical purposes. Our results provide important insights for understanding the bioactive protein differences in DM in different lactation stages.

## 1. Introduction

Donkey milk (DM) has special nutritional value and health care function. The dry matter, protein, fat, and lactose contents of DM are 9.53%, 1.57%, 1.16%, and 6.33%, respectively, which are closer to human milk (HM) compared to cow milk (CM) [1]. In addition, there are also a variety of functional nutritional factors, such as lysozyme, lactalbumin, lactoferrin, and immunoglobulin, in DM [2,3]. Compared with CM, DM can reduce calorie intake and the risk of diseases, such as hyperlipidemia, which is conducive to cardiovascular health [4,5]. DM is also an ideal substitute for infants with milk protein allergy [2].

Some studies have shown that DM has some important biological activities, such as antioxidant activity, immune stimulation, anti-inflammatory, antibacterial, metabolic disease prevention, and anticancer [4,6]. The in vitro study found that the addition of 500 μg/mL DM significantly improved the survival rate of damaged β cells [7]. The results of the in vivo study showed that after 4 weeks of DM treatment, the blood glucose of type 2 diabetic rats decreased, the body’s ability to scavenge free radicals and antioxidation level increased, and insulin resistance improved [7]. In addition, DM can treat diabetes by down-regulating phosphoenolpyruvate carboxykinase 1 and glucose-6-phosphatase [7]. Li et al. (2020) found that DM is rich in many known anticancer ingredients, which can inhibit the growth and metastasis of 4T1 triple-negative breast cancer in mice by inducing apoptosis [8]. Furthermore, DM can down-regulate the redox-sensitive inflammatory transcription factor NF of the skin fibroblasts-κ B pathway and activate the phosphorylated extracellular regulated protein kinase p-ERK pathway, indicating that DM plays a role in anti-inflammatory wound healing and cosmetic dermatology [9]. However, the corresponding components and mechanisms for DM to exert these biological activities are still unclear.

In order to clarify the bioactive proteins in DM and analyze their changes with the lactation stage, the differentially expressed proteins (DEPs) in DM at different lactation stages were first investigated by data-independent acquisition (DIA) proteomics analysis.

## 2. Materials and Methods

### 2.1. Milk Sample Collection and Preparation

The animal experiment in this study was approved by the Animal Care and Use Committee of Liaocheng University (Liaocheng, China) (2023022706). Milk samples were taken from 84 healthy Dezhou donkeys at 7 different lactation stages: 1 d (group A), 30 d (group B), 60 d (group C), 90 d (group D), 120 d (group E), 150 d (group F_), and 180 d (group G) after foaling (12 donkeys per stages), generally corresponding to colostrum (1 d), early (30 d and 60 d), middle (90 d and 120 d), and late (150 d and 180 d) lactation stages. The donkeys were housed on a farm in Dong’E, Liaocheng City, China. The donkeys were raised in a semi-closed house. All donkeys drank freely and were offered the same diet of grass hay (ad libitum) supplemented with 2 kg concentrate per head per day. The milk samples were collected during morning mechanical milking, frozen quickly in liquid nitrogen, and stored at −80 °C until analysis.

### 2.2. Total Protein Extraction

After thawing the milk samples, 3 individual milk samples from each lactation stage were combined into 1 sample. This results in 4 biological replicates per lactation stage. Next, centrifuge the milk samples at 14,000× *g* and 4 °C for 15 min to remove milk lipids and cell impurities. Some samples were taken into an ultrafiltration tube (15 mL, 10 kD) and centrifuged at 14,000× *g* for 15 min. The ultra-filtered sample was put into a centrifuge tube and balanced with Dissolved Buffer (8 M Urea, 100 mM TEAB, pH 8.5) (Sigma, St. Louis, MI, USA). Then, take the supernatant and add 1 M DTT (Sigma, St. Louis, MI, USA) to react at 56 °C for 1 h, and subsequently alkylate with sufficient iodoacetamide in the dark at room temperature for 1 h. The protein quantity was tested with a Bradford protein quantitative kit.

### 2.3. Trypsin Treatment

Dissolved Buffer was added to the protein sample to 100 μL. After that, trypsin (Promega, Madison, WI, USA) and a 100 mM TEAB buffer (Sigma, St. Louis, MI, USA) were added, and the sample was mixed and digested at 37 °C for 4 h. Next, add the trypsin and CaCl_2_ to digest overnight. Mix formic acid with the digested sample, adjust the pH to under 3, and centrifuge at 12,000× *g* for 5 min. Slowly load the supernatant onto a C18 desalination column, wash it three times with a washing buffer (0.1% formic acid, 3% acetonitrile), and then add an elution buffer (0.1% formic acid, 70% acetonitrile). Collect the eluents of each sample for lyophilization.

### 2.4. DDA Spectrum Library Construction

DDA spectrum library construction and DIA mode identification using UHPLC-MS/MS were performed.

#### 2.4.1. Separation of Fractions

**Figure 1:**
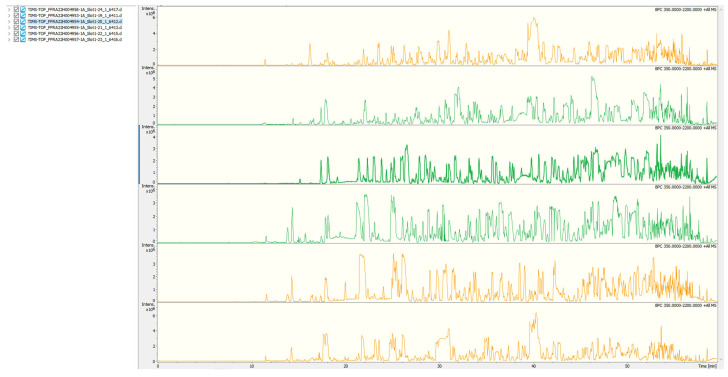
The chromatogram of the fractions collected.

Mobile phases A (2% acetonitrile, pH 10.0) and B (98% acetonitrile, pH 10.0) were used for gradient elution (Table 1 and Table 2). Dissolve the lyophilized powder (200 ng) in solution A and centrifuge at room temperature at 12,000× *g* for 10 min. Use C18 columns (Waters BEH C18, 4.6 × 250 mm, 5 μm) to fractionate the sample on the Rigol L3000 HPLC system with a column oven set at 45 °C. Monitor the eluates at UV 214 nm, collect 1 tube per minute, and finally combin into 4 or 6 fractions. The chromatogram of the fractions collected was shown in Figure 1. Dry all fractions under a vacuum and reconstruct them in 0.1% (*v*/*v*) formic acid (FA) in water.

#### 2.4.2. LC-MS/MS Analysis

The mobile phases A (100% water, 0.1% formic acid) and B (80% acetonitrile, 0.1% formic acid) were prepared. Dissolve the lyophilized powder in 10 μL solution A and centrifuge at room temperature at 14,000× *g* for 20 min. A total of 200 ng of supernatant was taken for detection. Shotgun proteomics analyses were performed using a nanoElute UHPLC (Bruker, Saarbrucken, Germany) system coupled with a Tims TOF pro2 (Bruker, Germany) mass spectrometer operating in data-dependent acquisition (DIA) mode in Novogene Co., Ltd. (Beijing, China). Peptides were separated in a homemade analytical column (25 cm × 75 μm, C-18 Silica Gel, 1.6 μm). The separated peptides were analyzed by Tims TOF pro2, with an ion source of captive spray and a spray voltage of 1.5 kV. The full scan range was from *m*/*z* 100 to 1700, the ramp time was 100 ms, and the lock duty cycle was 100%. The window size was 25 Da and the number of mobility windows was 2.

### 2.5. Data Analysis

#### 2.5.1. Identification and Quantitation of Protein

The resulting spectra were searched separately based on the 1417252-Equus_asinus_uniprot.fasta (34,696 sequences) database by the Spectronaut-Pulsar (Biognosys, Zurich, Switzerland) search engine. The search parameters were set as follows: mass tolerance for precursor ion was 10 ppm and mass tolerance for product ion was 0.02 Da. Carbamidomethyl was specified as a fixed modification, oxidation of methionine was specified as a dynamic modification, and acetylation was specified as an N-Terminal modification. A maximum of 2 missed cleavage sites were allowed.

In order to improve the analysis results quality, a Spectraut Pulsar further filtered the search results and identified Peptide Spectrum Matches (PSMs) with a reliability of over 99%. The identified protein contains at least 1 unique peptide. The identified PSMs and protein were retained and performed with FDR no more than 1.0%.

The DIA data was imported into Spectronaut 17 (Biognosys, Zurich, Switzerland) software to generate a DDA library, and ion-pair chromatographic peaks were extracted. The qualitative and quantitative measurements of peptides were achieved by matching the ion and calculating the peak area. Add iRT to the sample to correct retention time and set the cutoff of precursor ion Q value to 0.01. The quantitative results of protein were statistically analyzed using a *t*-test. The proteins whose quantitation was significantly different between experimental and control groups, (*p* < 0.05 and FC >± 1.5 (fold change, FC)) were defined as differentially expressed proteins (DEPs).

#### 2.5.2. Functional Analysis of Proteins

The interproscan program against the non-redundant protein database (including Pfam, PRINTS, ProDom, SMART, ProSite, PANTHER) was used in GO (Gene Ontology) and IPR (InterPro) functional analysis [10]. The protein family and pathway were analyzed using the COG (Clusters of Orthologous Groups) and KEGG (Kyoto Encyclopedia of Genes and Genomes) databases. DEPs were used for volcanic map analysis and enrichment analysis for GO, IPR, and KEGG [11].

## 3. Results

### 3.1. Characterization of Proteins in DM

**Figure 2:**
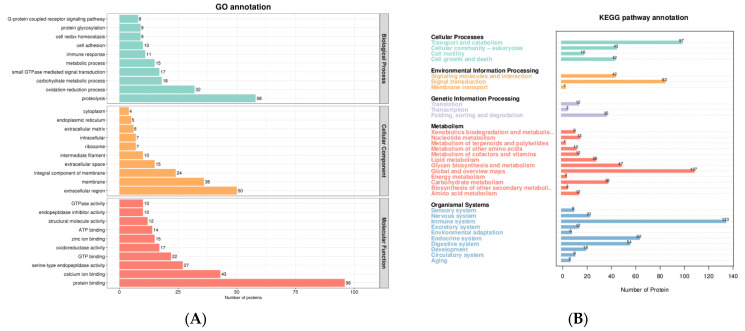
The identified protein functions were annotated by Gene Ontology analysis (**A**) and metabolic pathway analysis (**B**). Note: the horizontal ordinate was the protein number, while the vertical coordinate was the annotated GO (**A**) and KEGG (**B**) entries. Due to the excessive number of GO annotation results, the figure (**A**) only showed the top 10 results in each category. The green bar means Biological Process. The orange bar means Cellular Component. The red bar means Molecular Function.

In total, 4870 peptides and 805 proteins were characterized in DM (Table S1). Subcellular localization in DM mainly included extracell protein (243 proteins, 36.32%), plasma membrane protein (102 proteins, 15.25%), cytoplasm protein (74 proteins, 11.06%), lysosome protein (60 proteins, 8.97%), endoplasmic reticulum protein (58 proteins, 8.67%), and nucleus protein (51 proteins, 7.62%). The functions of identified proteins were annotated by GO analysis and metabolic pathway analysis (Figure 2). GO analysis revealed that these proteins were mainly related to biological processes (BP), such as proteolysis, the oxidation-reduction process, and the carbohydrate metabolic process; molecular functions (MF), such as protein binding, calcium ion binding, and serine-type endopeptidase activity; and cellular components (CC), such as extracellular region, membrane, and integral component of membrane. Metabolic pathway analysis suggested that the majority of the donkey proteins were associated with the immune system, global and overview maps, transport and catabolism, signal transduction, the endocrine system, and the digestive system.

### 3.2. Identification of DEPs in Different Lactation Stages

**Figure 3:**
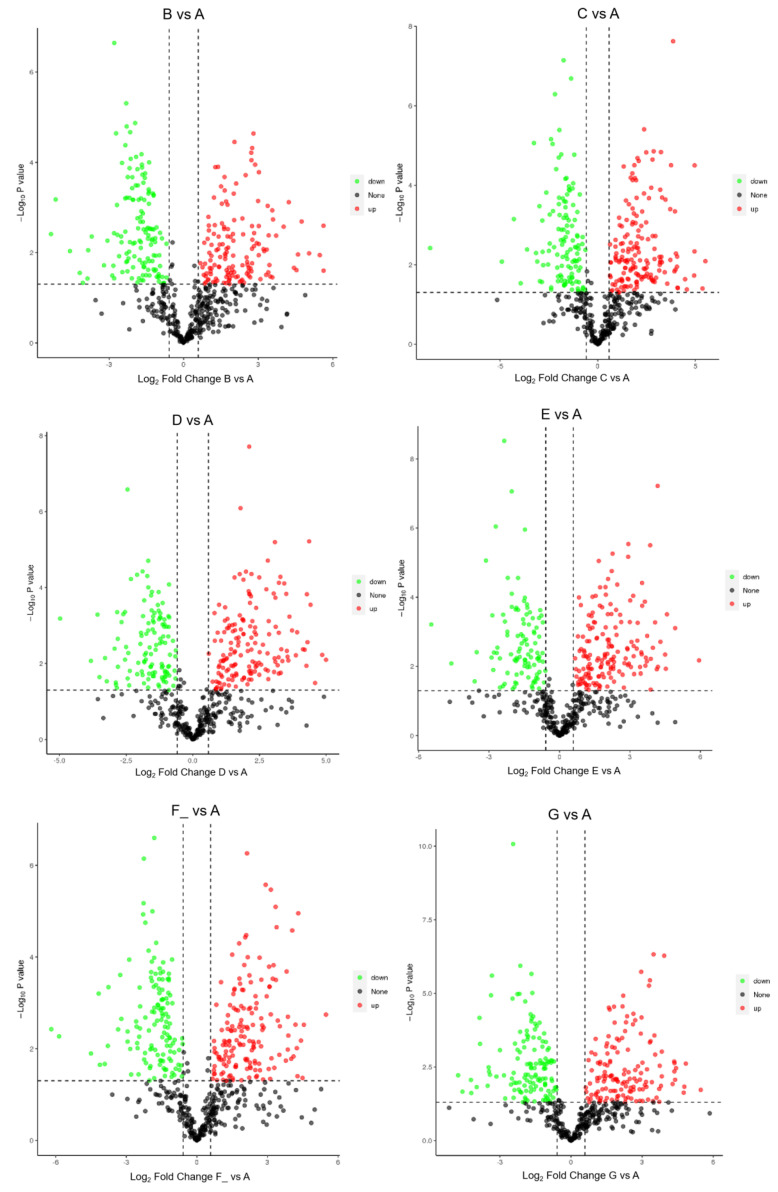
Volcano plots of DEPs in DM in different lactation stages using a data-independent acquisition (DIA)-based proteomics approach. FC = fold change. Note: milk samples were collected from Dezhou donkeys at seven different lactation stages: 1 d (A), 30 d (B), 60 d (C), 90 d (D), 120 d (E), 150 d (F_), and 180 d (G) after foaling.

**Figure 4:**
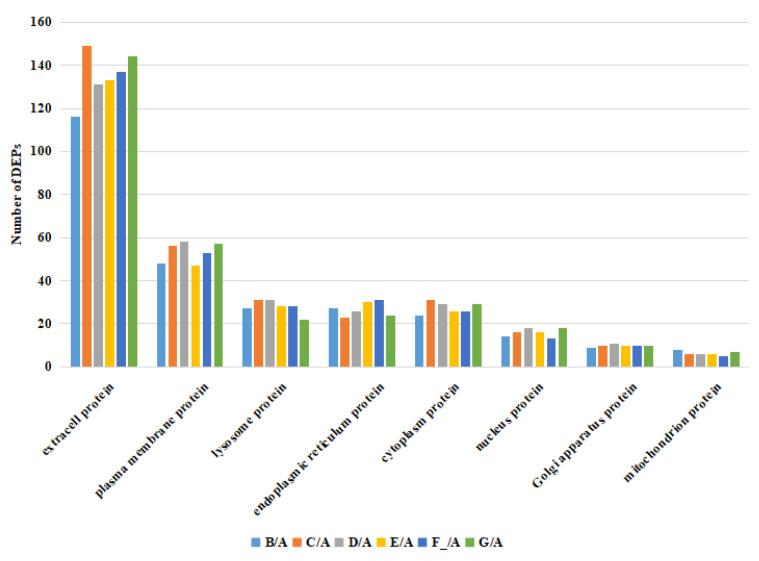
Subcellular localization of DEPs in the milk in different lactation stages. Note: milk samples were collected from Dezhou donkeys at seven different lactation stages: 1 d (A), 30 d (B), 60 d (C), 90 d (D), 120 d (E), 150 d (F_), and 180 d (G) after foaling.

The number of identified DEPs in DM in different lactation stages is shown in Table 3. The volcano plots of DEPs in milk from 1, 30, 60, 90, 150, and 180 d of dairy donkeys are shown in Figure 3. The DEPs in DM between different lactation stages are shown in Table S2. The subcellular localization of the DEPs is shown in Figure 4. Taking the B vs. A comparison group as an example, a total of 327 candidate DEPs, including 161 up- and 166 down-regulated DEPs, were obtained from the GO and metabolic pathway analysis.

**Figure 5:**
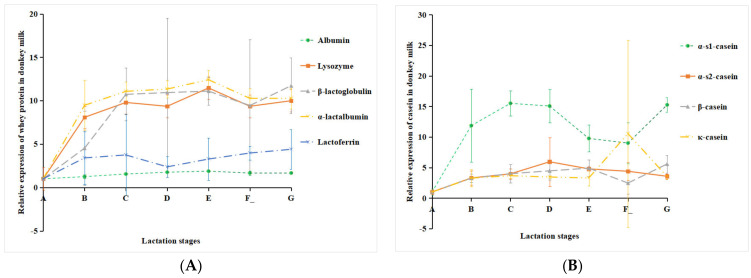
The expression levels of whey protein (**A**) and casein (**B**) in DM in different lactation stages related to colostrum. Note: milk samples were collected from Dezhou donkeys at seven different lactation stages: 1 d (A), 30 d (B), 60 d (C), 90 d (D), 120 d (E), 150 d (F_), and 180 d (G) after foaling.

The relative expression of DM proteins (whey protein and casein) in different lactation stages was analyzed. The results showed that whey protein in DM mainly included α-lactalbumin, β-lactoglobulin, albumin, lysozyme, and lactoferrin, and casein mainly included κ-casein, β-casein, α-s1-casein, and α-s2-casein. The expression level of all the whey proteins and casein in mature milk (groups B, C, D, E, F_, and G) was higher than colostrum (group A) (Figure 5). With the extension of the lactation period, the relative expression level of lactoferrin increased. The relative expression levels of other whey proteins (albumin, lysozyme, β-lactoglobulin, and α-lactalbumin) gradually increased within 60 days of lactation and then reached the plateau stage (Figure 5A). As shown in Figure 5B, the level of α-s1-casein increased in the early lactation stage, decreased in the middle to late lactation stages, and then increased again on the 180th day of lactation (Figure 5B). The relative expression level of α-s2-casein and κ-casein increased and then decreased with the extension of the lactation period. β-casein gradually increased within 30 days of lactation and then remained stable (Figure 5B).

### 3.3. GO Analysis of DEPs

The DEPs between the different lactation stages in DM were classified into CC, MF, and BP, according to GO annotation terms. Take B. vs. A, for example. GO analysis revealed that the 327 DEPs were mainly related to BP, such as blood coagulation, hyaluronan metabolic process, and the single multicellular organism process; and MF, such as endopeptidase activity and serine-type endopeptidase activity (Table 4).

### 3.4. Metabolic Pathway Analysis of DEPs

**Figure 6:**
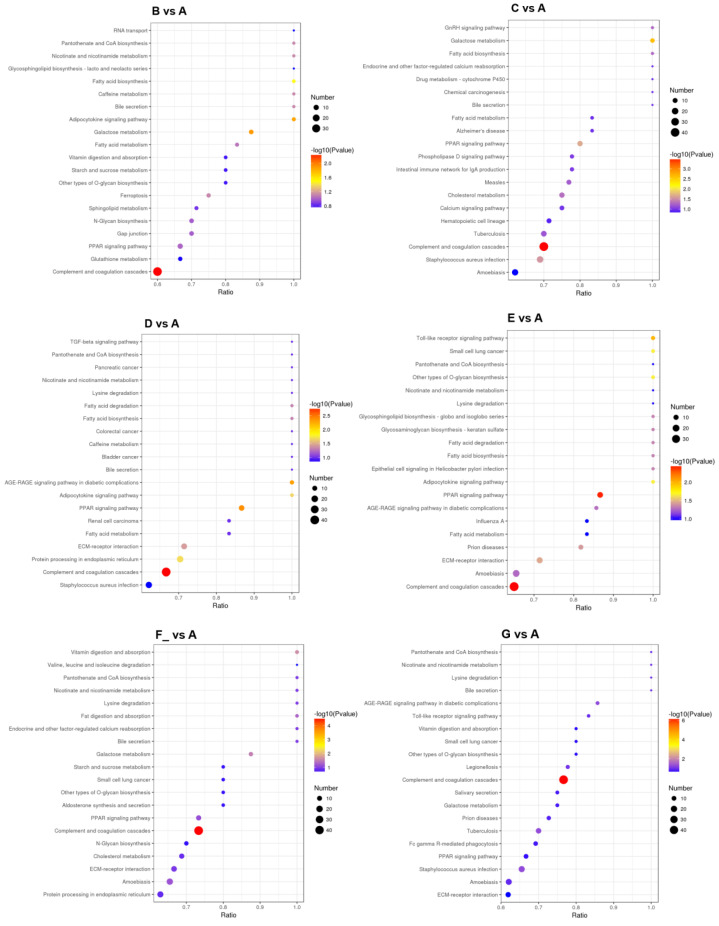
The top 20 main metabolic pathways associated with DEPs Note: milk samples were collected from Dezhou donkeys at seven different lactation stages: 1 d (A), 30 d (B), 60 d (C), 90 d (D), 120 d (E), 150 d (F_), and 180 d (G) after foaling.

The top 20 main metabolic pathways associated with the DEPs are shown in Figure 6. Take B vs. A, for example. Metabolic pathway analysis showed that most down-regulated DEPs were related to complement and coagulation cascades, and up-regulated DEPs were involved in galactose metabolism, the adipocytokine signaling pathway, and fatty acid biosynthesis (Table 5).

## 4. Discussion

The proteins in DM are increasingly attracting researchers’ interest due to their diverse biological activities. In this study, the proteins in DM from 1 d to 180 d were first investigated using a DIA proteomic analysis. There were 805 proteins in DM, including the main whey protein (α-lactalbumin, β-lactoglobulin, albumin, lysozyme, and lactoferrin) and the casein (κ-casein, β-casein, α-s1-casein, and α-s2-casein). These proteins were mainly related to BP, such as proteolysis, the oxidation-reduction process, and the carbohydrate metabolic process; MF, such as protein binding, calcium ion binding, and serine-type endopeptidase activity; and CC, such as extracellular region, the membrane, and the integral component of the membrane. The majority of the DM proteins were associated with the immune system, global and overview maps, transport and catabolism, signal transduction, the endocrine system, and the digestive system.

The content and composition of milk protein may vary among species, breeds, lactation stages, diets, and seasons [12,13,14]. Some studies have shown that as the lactation period prolongs, the milk protein content in DM gradually decreases [15,16]. It was reported that DM protein content was significantly affected by the lactation stage, except for the caseins [17]. Another study suggested that the macronutrient content in mature milk remains stable throughout the lactation period [18]. However, in this study, the relative expression levels of casein (κ-casein, β-casein, α-s1-casein, and α-s2-casein) increased and then decreased with the extension of the lactation period. The contents of the main whey proteins in DM were also affected by the lactation stage. The relative expression level of lactoferrin increased from 1 d to 180 d and the relative expression levels of albumin, lysozyme, β-lactoglobulin, and α-lactalbumin gradually increased within 60 d; after that, it remained constant. Whey proteins represented 58% of total DM protein [17]. The whey proteins play an important physiological role in preventing milk allergy and antibacterial and immune regulation [6]. These changes in DM whey protein content during lactation ensure the biological activities, such as antibacterial and immune regulation, of mature milk, which are conducive to the intestinal health and growth of donkey foal.

Milk can provide nutrients and bioactive substances for newborns. Milk is divided into colostrum (1–3 days postpartum) and mature milk (10 days postpartum). The composition of milk changes with the lactation period. Colostrum is characterized by high energy and immunoglobulin G content. Compared to colostrum, the macronutrient content in mature milk remains stable throughout the lactation period [18]. Li et al. (2019) analyzed the metabolites in DM (colostrum and mature milk) with untargeted metabolomics, and 270 metabolites were characterized in total [19]. Fifty-two differential metabolites in the colostrum were found, which were involved in thirty-two metabolic pathways, such as lysine biosynthesis, purine metabolism, fatty acid elongation in mitochondria, and the citrate cycle [19].

In this study, there were 445 DEPs between the donkey mature milk (30–180 d) and colostrum (1 d), with 219 down-regulated proteins and 226 up-regulated proteins. The down-regulated 219 DEPs were mainly related to immunity and disease resistance metabolic pathways, such as complement and coagulation cascades, staphylococcus aureus infection, systemic lupus erythematosus, prion diseases, AGE-RAGE signaling pathways in diabetic complications, and pertussis. DEPs with a fold change greater than five mainly included complement factor D, complement C8, mannose-binding lectin 2, Ig-like domain-containing protein, and interleukin 1 receptor accessory protein. These results indicated that donkey colostrum had a unique advantage in immunoregulation and disease resistance.

The up-regulated 226 DEPs were mainly involved in metabolic pathways related to nutrient metabolism, such as fatty acid metabolism, sphingolipid metabolism, adipocytokine signaling pathways, bile secretion (fat metabolism), amino sugar and nucleotide sugar metabolism, galactose metabolism (carbohydrate metabolism), protein processing in endoplasmic reticulum, N-Glycan biosynthesis (nucleic acid metabolism), purine and pyrimidine metabolism (nucleic acid metabolism), and nicotinate and nicotinamide metabolism (vitamin metabolism). For example, the long-chain fatty acid CoA ligase 1 (ACSL1) (FC > 45), perilipin 2 (FC > 13), and sphingosine kinase 1 (SphK1, FC > 12) were highly expressed in milk in early and mid-lactation. ACSL1 can catalyze fatty acid conversion into acyl-CoA, which can undergo either β-oxidation or re-esterification, depending on physiological conditions and hormonal signaling [20,21]. Perilipin 2 mainly participates in the formation of lipid droplets in milk [22,23]. SphK1 can catalyze the phosphorylation of sphingosine to produce sphingosine 1-phosphate, which can promote cell proliferation and angiogenesis and participate in the process of immune regulation and inflammation [24,25]. The high expression of these proteins can ensure the healthy development of the donkey foal’s intestines and the absorption and utilization of nutrients, such as milk fat.

Moreover, there were also some other DEPs in mature milk from early (30–60 d), mid- (90–120 d), and late (150–180 d) lactation stages related to nutrition, transportation, cell proliferation and differentiation, antibacterial, anti-inflammatory, and cosmetic effects.

### 4.1. Nutrition and Transportation

There was more selenoprotein F (FC > 6) in mature milk in the mid and late lactation (90–180 d) stage. Selenium (Se) is one important essential trace element in humans and animals. It is a structural component with many proteins and plays a crucial role in physiological processes such as DNA synthesis, the scavenging of toxins, antioxidant defense, and thyroid hormone metabolism [26,27]. Se deficiency can cause reproductive disorders (such as abortion and infertility) and muscular diseases (such as white muscle disease) [27]. However, excessive Se can cause animal poisoning [28]. Compared to inorganic Se, organic Se is considered a safe and effective bioavailable source for humans and animals [27,29]. Zhang et al. (2020) studied the hypotensive and immune-enhancing components in buffalo whey at different altitudes [30]. It was found that the selenoprotein F content of whey at low altitudes was higher than whey at high altitudes, and buffalo milk at low altitudes was suitable for producing immune-regulation milk [30]. DM also had a high content of Se, which was beneficial for normal immune system function [31,32]. The result of this study showed that DM from mid and late lactation had a high content of selenoprotein F. Therefore, DM from mid and late lactation can serve as a good source of organic Se supplementation.

In addition, the contents of some transporters, such as transcobalamin 1 (TC, FC > 26) and solute carrier family 36 member 1 (SLC36A1, FC > 19), were also high in mature milk (30–180 d). Milk is an abundant source of bioavailable vitamin B_12_ (VB_12_). However, the bioavailability of VB_12_ in milk is influenced by many factors, such as transport carriers and intestinal uptake capacity. TC is a VB_12_-specific binding protein, which may influence VB_12_ bioavailability. Fedosov et al. (2019) investigated the effects of vitamin carriers on VB_12_ nutritional availability in cow and buffalo milk and found that VB_12_ bioavailability was higher in CM (with TC-VB_12_) than buffalo milk (with haptocorrin-VB_12_) [33]. The research by Hine et al. (2014) found that bovine milk TC can stimulate bovine, mouse, and human intestine VB_12_ uptake [34]. The TC-VB_12_ complex extracted from milk can be used as a natural biological source for VB_12_ to overcome the malabsorption of VB_12_ [34]. Our study found that donkey mature milk (30 d–180 d) contained abundant TC. In terms of the bioavailability of VB_12_, DM and CM were more beneficial for human health [35]. SLC36A1 is a neutral amino acid transporter. It exerts a variety of important physiological functions, including promoting nutrient absorption, controlling nutrient recycling, and activating the mTORC1 signaling pathway [36]. In a study by Wang et al. (2021), SLC36A1 mediated the activation of mTORC1 induced by leucine [37]. Therefore, SLC36A1 in DM may play a role in the nutrient’s absorption and signal transduction, which requires further research.

### 4.2. Cell Proliferation and Differentiation

There were also some cell proliferation and differentiation associated components, such as transforming growth factor-beta-induced protein ig-h3 (beta ig-h3, FC > 24), sushi repeat-containing protein X-linked (SRPX, FC > 22), secreted phosphoprotein 1 (SPP1, FC > 7), tyrosine–protein kinase (FC > 11), and milk fat globule-EGF factor 8 protein (Mfge8, FC > 5) in mature milk (30–180 d). Among them, beta ig-h3, SRPX, and SPP1 were extracellular matrix-associated proteins, while tyrosine–protein kinase and Mfge8 played a role in cell proliferation, differentiation, and apoptosis.

Beta ig-h3 is an extracellular matrix protein that exists in many tissues, such as the kidneys, spleen, bladder, and lungs, and can be detected in culture media of human lung and bladder smooth muscle cells and fibroblasts cultured in vitro [38]. Beta ig-h3 is involved in the cell’s adhesion, migration, proliferation, differentiation, and apoptosis [39,40]. SRPX is a chondroitin sulfate proteoglyean that belongs to extracellular matrix protein. It can promote synaptogenesis in the cerebral cortex, regulate the development of neurons, and regulate the immune system [41]. SPP1 is a multifunctional protein that plays a role in intercellular communication and extracellular matrix, with functions of cytokines, chemokines, and signal transduction [42]. It plays an important role in disease (cardiovascular disease, cancer, diabetes, and kidney stone disease), inflammation, and wound healing processes [43]. The presence of these extracellular matrix-related proteins in DM was beneficial for the proliferation and differentiation of intestinal cells in donkey foals and infants.

Tyrosine–protein kinases can phosphorylate the protein tyrosine residues and play a very important role in cell growth and differentiation. The bioactive substances in HM had protective effects on infants. HM can induce the proliferation of fetal small intestine cells, which depend on the signaling pathway involved in tyrosine–protein kinase [44]. In addition, tyrosine–protein kinase also collaborates with Mfge8 to participate in the clearance of apoptotic cells. Mfge8 can eliminate apoptotic cells by regulating phagocytes and plays an important role in self-stabilization, angiogenesis, inflammation, and other processes. It was reported that tyrosine kinase and Mfge8 were up-regulated during inflammation and synergistically cleared damaged cells, which was beneficial for repairing dysfunctional tissues [45]. Thus, a high expression of tyrosine–protein kinase and Mfge8 in DM may promote the growth and proliferation of small intestine cells and repair damaged intestinal tissue, which is beneficial for the intestinal function of donkey foals and infants.

### 4.3. Antibacterial and Anti-Inflammatory Activity

There were some enzymes such as alpha-mannosidase (FC > 18), alpha-1,2-Mannosidase (FC > 8), Peroxiredoxin 4 (Prdx4, FC > 17), cathepsin B (FC > 10), and lysozyme (FC > 9) in donkey mature milk (30–180 d), which played important roles in antibacterial and anti-inflammatory activity. Alpha-mannosidases are widely expressed in human and animal tissues and body fluids [46]. They play an important role in glycoproteins metabolism, such as protein glycosylation and hydrolysis of glycoproteins. Alpha-mannosidases were mainly involved in the folding, maturation, transportation, and biological activity of newly formed glycoproteins, and thus play a role in cell adhesion, inflammatory response, and immune monitoring [47]. It was reported that alpha-mannosidase digested the biofilm and allowed neutrophils to kill the bacteria in infected mice [48]. Prdx4 is a vital antioxidant in cells that can hydrolyze hydrogen peroxide and promote oxidative protein folding [49]. Prdx4 plays a critical role in inflammatory diseases. It was reported that Prdx4 prevented inflammation and cell apoptosis by inhibiting oxidative stress [50]. A study by Yamada et al. (2012) found that the overexpression of human Prdx4 in transgenic mice can protect the pancreatic beta-cells from damage (insulitis) by inhibiting oxidative stress and inflammatory signals [51]. Cathepsin B is a lysosomal cysteine protease. It plays a role in protein catabolism and may participate in some physiological processes such as antigen processing and presentation, hormone activation, apoptosis, aging, autophagy, and bone turnover [52,53]. In addition, a lysozyme is a kind of hydrolase that can hydrolyze peptidoglycan, the main component of bacterial cell walls. Proteomic analysis of DM showed that lysozyme had antibacterial activity [6]. It was found that the oral administration of heat-treated DM with preserved lysozyme activity can improve intestinal injury induced by chronic stress in mice [54]. The results of other studies show that a lysozyme, together with lactoferrin and immunoglobulin, can inhibit the growth of gastrointestinal pathogenic microorganisms and reduce the incidence of gastrointestinal infection in infants [12]. These results indicated that DM had potential biological activities, such as antioxidant activity, immune stimulation, anti-inflammatory, and antibacterial properties (Figure 2 and Figure 6). DM is an ideal source of nutrition for growing children, convalescent patients, and the elderly [4,6].

### 4.4. Cosmetics

DM has a long history of being used for cosmetic and medical purposes. A study by Kocic et al. (2020) found that the cream made from DM enhanced the moisture of the skin, increased the penetration of phospholipids and essential proteins, and may also have anti-aging effects [9]. In this study, the collagen type XV alpha 1 chain (FC > 9) in mature milk (30–180 d) was higher than colostrum. Collagen type XV is a proteoglycan located in the basement membrane of endothelial and epithelial cells. It was reported that collagen can inhibit the production of melanin, maintain the moisture content of the stratum corneum and the integrity of the fiber structure, and have whitening, moisturizing, and wrinkle-removing effects on the skin [55]. The results in a study by Li et al. (2023) indicated that DM can inhibit the production of melanin, reduce UVB-induced damage, and restore skin barriers, thus having whitening and anti-photoaging effects [56]. Collagen type XV may be an important component with cosmetic functions in DM. Furthermore, some studies found that mice lacking collagen type XV exhibited skeletal myopathy, cardiovascular defects, and cardiomyopathy [57,58]. In addition, collagen XV reduced the inflammation induced by renal damage in mice [59]. Some studies showed that collagen type XV was involved in Dupuytren’s contracture (inherited connective tissue disease of humans) and osteoblast differentiation and mineralization [60,61]. These results indicated that collagen type XV in DM could play an important role in anti-inflammatory and disease resistance.

## 5. Conclusions

In this study, the DEPs in DM in different lactation stages were first investigated by DIA proteomics analysis. A total of 805 proteins were identified. The majority of the DM proteins were associated with the immune system, global and overview maps, transport and catabolism, signal transduction, the endocrine system, and the digestive system. The composition and content of milk proteins varied with the lactation stage. DEPs in colostrum were related to complement and coagulation cascades, and the DEPs in mature DM were involved in galactose metabolism, adipocytokine signaling pathways, and fatty acid biosynthesis. DM had a high content of Se and transcobalamin 1, which was a good source of organic Se (mid and late lactation) and VB_12_ for humans. In addition, some DEPs could explain different biological activities, such as promoting cell growth and proliferation, and having antioxidant activity, immune stimulation, anti-inflammatory, antibacterial, and anti-aging effects. These results suggested that DM could be an ideal source of nutrition for growing children, convalescent patients, and the elderly.

## Figures and Tables

**Table 1:** Four of peptide fractions separation liquid chromatography elution gradient table.

Time (min)	Flow Rate (mL/min)	Mobile Phase A (%)	Mobile Phase B (%)
0	1	97	3
10	1	95	5
20	1	80	20
27	1	60	40
29	1	50	50
30	1	30	70
35	1	0	100

**Table 2:** Six of peptide fractions separation liquid chromatography elution gradient table.

Time (min)	Flow Rate (mL/min)	Mobile Phase A (%)	Mobile Phase B (%)
0	1	97	3
10	1	95	5
12	1	90	10
13	1	85	15
50	1	60	40
53	1	40	60
54	1	0	100
58	1	0	0
60	1	0	0
70	1	0	0

**Table 3:** The number of DEPs in DM in different lactation stages.

Compared Samples	Number of DEPs	Regulated Type	Fold Change > 1.5
B vs. A	620	Up-regulated	161
		Down-regulated	166
C vs. A	622	Up-regulated	178
		Down-regulated	201
D vs. A	611	Up-regulated	176
		Down-regulated	188
E vs. A	604	Up-regulated	177
		Down-regulated	185
F_ vs. A	620	Up-regulated	176
		Down-regulated	191
G vs. A	623	Up-regulated	157
		Down-regulated	216
B, C, D, E, F_, G vs. A	805	Up-regulated	226
		Down-regulated	219

Note: milk samples were collected from Dezhou donkeys at seven different lactation stages: 1 d (A), 30 d (B), 60 d (C), 90 d (D), 120 d (E), 150 d (F_), and 180 d (G) after foaling.

**Table 4:** GO analysis of the DEPs between group B and group A.

GO Class	Up-Regulated	Down-Regulated
Molecular functions	Serine-type endopeptidase activity	Small molecule binding
	Endopeptidase activity	Guanyl nucleotide binding
	Serine-type peptidase activity	Purine nucleotide binding
	Peptidase activity	Nucleotide binding
	Acting on L-amino acid peptides	Organic cyclic compound binding
	Endopeptidase inhibitor activity	Heterocyclic compound binding
	Serine-type endopeptidase inhibitor activity	Purine ribonucleoside binding
	Lipid binding	Purine ribonucleotide binding
	Thiol oxidase activity	Purine ribonucleoside triphosphate binding
		GTP binding
		Carbohydrate derivative binding
Biological process	Macromolecule metabolic process	
	Blood coagulation, proteolysis	
	Hyaluronan metabolic process	
	Glycosaminoglycan metabolic process	
	Single multicellular organism process	
	Lipoprotein metabolic process	
	Platelet activation	
	Protein metabolic process	
	Metabolic process	
	Lipid transport	
	Organic substance metabolic process	
	Response to stress	
	Macromolecule localization	
Cellular component	Extracellular region	
	Extracellular region part	
	Extracellular space	
	Fibrinogen complex	

**Table 5:** Metabolic pathway analysis of the DEPs between group B and group A.

Regulated Type	Metabolic Pathway
Up-regulated	Fatty acid biosynthesis
	Fatty acid metabolism
	Sphingolipid metabolism
	Protein processing in the endoplasmic reticulum
	Adipocytokine signaling pathway
	Peroxisome
	Caffeine metabolism
	Nicotinate and nicotinamide metabolism
	Bile secretion
	Purine metabolism
	Galactose metabolism
	Fatty acid degradation
	Platinum drug resistance
	Amino sugar and nucleotide sugar metabolism
	Long-term depression
	N-glycan biosynthesis
	RNA transport
Down-regulated	Complement and coagulation cascades
	Staphylococcus aureus infection
	Systemic lupus erythematosus
	Glycosaminoglycan biosynthesis—keratan sulfate
	Neuroactive ligand–receptor interaction
	Glycosphingolipid biosynthesis—lacto and neolacto series

## Data Availability

Data is contained within the article or Supplementary Material.

## Supplementary Materials

The following supporting information can be downloaded at: https://www.mdpi.com/article/10.3390/foods12244466/s1. Table S1: The proteins characterized in DM, Table S2: The DEPs in DM between different lactation stages.
